# Exploratory analysis of morphology–hemodynamics–perfusion relationships after intracranial stenting in severe intracranial atherosclerotic stenosis

**DOI:** 10.3389/fneur.2026.1900989

**Published:** 2026-09-07

**Authors:** Xulong Yin, Zhen Li, Fuping Huang, Yuxiao Chen, Ting Hong, Yi You, Rong Fu, Yusheng Zhao, Huiru Shen, Hui Wang, Qi Fang

**Affiliations:** 1 Department of Neurology, The First Affiliated Hospital of Soochow University, Suzhou, Jiangsu, China; 2 Institute of Stroke Research, Soochow University, Suzhou, Jiangsu, China

**Keywords:** computational fluid dynamics, CT perfusion, hemodynamic, intracranial atherosclerotic stenosis, stent implantation

## Abstract

**Background:**

Intracranial artery stenting is widely used to treat severe intracranial atherosclerotic stenosis (ICAS). However, the relationships among post-stenting morphological remodeling, hemodynamic recovery, and perfusion imaging changes remain unclear.

**Methods:**

This retrospective single-center study included 131 patients with severe ICAS (70–99%) who underwent intracranial stenting. Vascular morphology was assessed by digital subtraction angiography, hemodynamics were quantified using computational fluid dynamics, and CT perfusion was used to evaluate perfusion imaging changes. Associations among morphological, hemodynamic, and perfusion changes were analyzed using correlation, multivariable, and mediation analyses.

**Results:**

A total of 131 patients were analyzed, including 108 with anterior circulation lesions (77 males; 71.3%; age 65.50 years [57.00–69.00]). Stenting significantly improved luminal morphology, hemodynamic parameters, and CT perfusion metrics (all *p* < 0.05). After adjustment for hemodynamic changes, morphological changes were no longer independently associated with perfusion imaging changes. Changes in pressure ratio (ΔPR) showed the strongest associations with reductions in Tmax-based perfusion-delay volume, nonlinear threshold effects, and a significant mediating effect on the association between morphological remodeling and improvement in perfusion-delay metrics. These associations were more evident in the anterior circulation than in the posterior circulation.

**Conclusion:**

Changes in translesional pressure ratio were more closely associated with post-stenting improvement in perfusion-delay metrics than geometric stenosis measures alone, suggesting that pressure ratio may serve as an intermediate hemodynamic marker linking anatomical remodeling with downstream perfusion imaging changes.

## Graphical abstract

**Figure:**
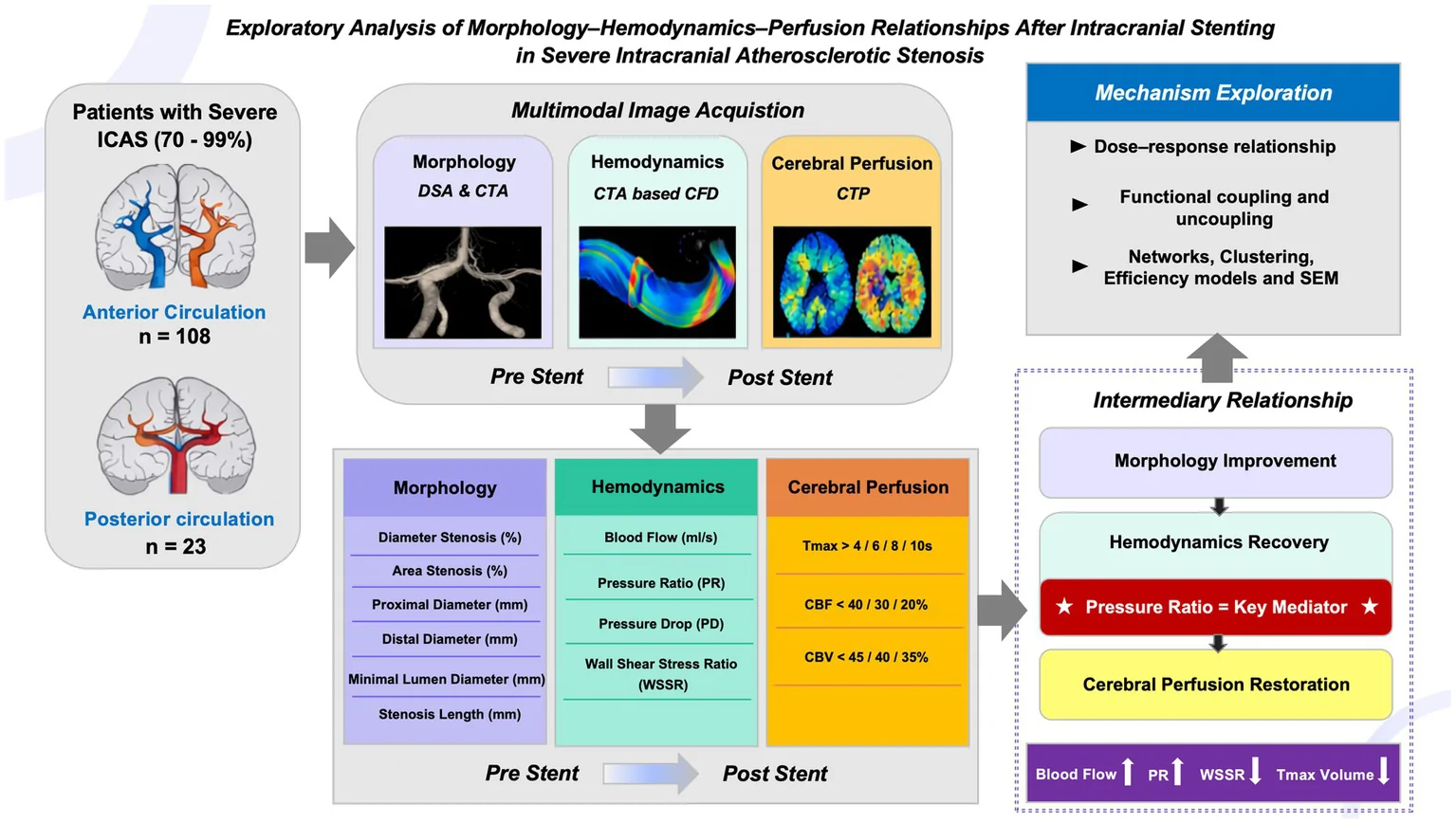

## 1 Introduction

Intracranial atherosclerotic stenosis (ICAS) is a major cause of ischemic stroke, accounting for 30–50% of ischemic stroke cases in Asian populations (1). Intracranial artery stenting is employed in patients with symptomatic ICAS who are refractory to aggressive medical therapy, with the aim of improving distal cerebral perfusion and reducing the risk of recurrent stroke (2). Although endovascular treatment can effectively relieve luminal narrowing, uncertainty persists regarding the extent to which post-stenting anatomical reconstruction translates into hemodynamic improvement and downstream perfusion changes (3–5).

Traditional methods such as computed tomography angiography (CTA) or digital subtraction angiography (DSA) focus on residual stenosis or geometric remodeling, but these morphological indicators do not fully capture complex hemodynamic changes (6). Several studies have confirmed the feasibility of hemodynamic analysis in the study of ICAS, and many related hemodynamic indicators, such as pressure ratio (PR), wall shear stress (WSS), fractional pressure ratio (FPR), quantitative blood flow ratio (QFR), and conductive pressure differential have been used as reference indicators (7–10). Computational fluid dynamics (CFD) can quantitatively describe hemodynamic changes and their effects in ICAS, providing a visual basis for analyzing post-treatment arterial blood flow restoration and energy loss (11–13). However, CFD alone cannot fully characterize downstream perfusion-related imaging changes after stenting.

Computed tomography perfusion (CTP) provides imaging-based perfusion information through parameters such as cerebral blood flow (CBF), cerebral blood volume (CBV), and time to maximum (Tmax) (14, 15). In the current chronic intracranial atherosclerotic stenosis (ICAS) setting, these metrics can complement vascular and hemodynamic assessments by characterizing downstream perfusion-related abnormalities, with Tmax primarily reflecting perfusion delay rather than direct tissue viability. Although previous studies have combined CTP and hemodynamic assessment in ICAS (10, 16), few have systematically integrated morphological, computational fluid dynamics (CFD)-derived hemodynamic, and perfusion imaging parameters to evaluate their relationships before and after intracranial stenting. Consequently, the association among vascular remodeling, hemodynamic changes, and perfusion imaging changes remains insufficiently understood.

Therefore, this study aimed to explore the associations among post-stenting vascular morphology, hemodynamic changes derived from computational fluid dynamics (CFD), and CT perfusion metrics in patients with severe intracranial atherosclerotic stenosis (ICAS). In particular, we examined whether post-stenting morphological remodeling was associated with downstream perfusion imaging changes through hemodynamic changes, especially changes in translesional pressure ratio. We also evaluated whether these associations differed across vascular territories and across different levels of perfusion abnormality.

## 2 Materials and methods

### 2.1 Study population and clinical data collection

This retrospective study was approved by the Ethics Committee of the First Affiliated Hospital of Soochow University (approval no. 2024733). The requirement for written informed consent was waived due to the retrospective design and anonymized analysis of routinely collected data. This study retrospectively included patients with ICAS who underwent intracranial arterial stenting at the First Affiliated Hospital of Soochow University between January 2021 and December 2024. Inclusion criteria were as follows: (1) hospitalized patients aged ≥18 years with intracranial atherosclerotic stenosis of severe degree (stenosis ranging from 70 to 99%), involving the intracranial internal carotid artery, middle cerebral artery, vertebral artery V4 segment, or basilar artery; (2) all patients underwent intracranial arterial stenting; (3) all patients underwent both CTA and CTP, and successfully constructed CFD models based on CTA; (4) all patients underwent DSA. Exclusion criteria included ischemic stroke due to non-atherosclerotic intracranial stenosis such as moyamoya disease, vasculitis, or dissection; complete occlusion of intracranial arteries; coexisting underlying cardiac diseases such as atrial fibrillation. Baseline demographic and clinical data included age, sex, vascular territory (anterior vs. posterior circulation), blood pressure, metabolic indices, and major vascular risk factors. Neurological status was assessed on admission using the National Institutes of Health Stroke Scale and at discharge using the modified Rankin Scale.

### 2.2 Multimodal imaging and CTA-based hemodynamic analysis

All included patients underwent multimodal imaging as part of the routine pre- and post-stenting evaluation, including non-contrast CT, CTA, CTP, and DSA. For each patient, preprocedural DSA and CTA/CTP were acquired within 7 days before stent placement. Follow-up DSA and CTA/CTP were performed approximately 6 months after stenting. DSA and CTA/CTP were not necessarily acquired on the same calendar day but were scheduled within the same preprocedural or follow-up assessment window. The CTA data used for CFD reconstruction and the CTP data used for perfusion analysis were obtained during the same CTA/CTP examination. Accordingly, all post-stenting comparisons in this study represent medium-term imaging changes at approximately 6 months rather than intraoperative, immediate, 24-h, or 48-h effects. Imaging was retrospectively collected from clinically available pre- and post-procedural examinations. CT imaging was performed using a 256-slice GE Revolution CT scanner (GE Healthcare, Milwaukee, USA). CTP datasets were processed using CareStroke software (Shukun Technology, Beijing, China) to generate maps of time to maximum (Tmax), cerebral blood flow, and cerebral blood volume with delay and dispersion correction. In the present chronic ICAS setting, Tmax thresholds were interpreted as imaging markers of perfusion delay rather than direct surrogates of tissue viability, whereas CBF- and CBV-based thresholds were treated as perfusion abnormality metrics. DSA was performed using either a Siemens Artis Zeego or Toshiba INFX-8000 V system via femoral access. Intravenous heparin (2000 units) was administered before angiography, and iodixanol contrast medium (320 mg/mL) was injected at 4–5 mL/s (6–8 mL total). CTA spatial resolution was approximately 0.37 mm. CTA-based CFD models were constructed using ANSYS software. Three-dimensional arterial geometries were reconstructed from CTA images and discretized with 0.5–1 million elements. Inlet boundary pressure was assigned using predefined pressure values derived from previous studies and supported by the published literature. Outlet pressure was calculated using AccuFFicas software (ArteryFlow Technology, Hangzhou, China) (17). Vessel walls were assumed rigid with no-slip conditions, and blood was modeled as an incompressible Newtonian fluid (density: 1,050 kg·m^−3^; viscosity: 0.0035 kg·m^−1^·s^−1^) (18, 19). Blood flow was simulated by solving the Navier–Stokes equations.

### 2.3 Definition of relevant parameters

Morphological analysis included six parameters: diameter stenosis% (DS%) (20), area stenosis% (AS%), proximal luminal diameter at the stenosis, distal diameter at the stenosis, minimum lumen diameter (21), and stenosis length. Hemodynamic assessment included four parameters: flow rate in the stenosis-bearing artery, pressure drop (PD), pressure ratio (PR), and wall shear stress ratio (WSSR). PD was defined as the pressure proximal to the stenosis minus the pressure distal to the stenosis. PR was defined as the ratio of mean distal pressure to mean proximal pressure (P_distal_/P_proximal_) (7, 18). WSSR was defined as the ratio of mean wall shear stress at the stenotic throat to that at the proximal reference segment (18, 22, 23). For all Δ variables, the post-stenting value referred to the approximately 6-month follow-up examination.

### 2.4 Statistical analyses

Continuous variables were assessed for normality using the Shapiro–Wilk test and were presented as mean ± standard deviation or median (interquartile range), as appropriate. Paired pre- and post-stenting comparisons were performed using the paired t test or Wilcoxon signed-rank test, as appropriate, whereas between-group comparisons were performed using the independent-samples t test or Mann–Whitney U test. Categorical variables were presented as counts and percentages and were compared using the chi-square test or Fisher’s exact test, as appropriate. Changes in morphological, hemodynamic, and perfusion parameters were quantified as Δ values (post-stenting minus pre-stenting) and compared between anterior and posterior circulation groups. Standardized effect sizes were calculated to summarize the magnitude of post-stenting changes. Associations among morphological, hemodynamic, and perfusion variables were evaluated using correlation analyses, multivariable regression, spline-based models, and bootstrap mediation analyses (5,000 resamples). Additional exploratory analyses were described in the Supplementary Methods. Variables with highly zero-inflated or degenerate distributions were interpreted cautiously, and nominal *p* values from rank-based paired tests were not considered sufficient evidence of clinically meaningful change. All tests were two-sided, and *p* < 0.05 was considered statistically significant. Statistical analyses were performed using Python (version 3.10).

## 3 Results

### 3.1 Baseline characteristics

**Figure 1:**
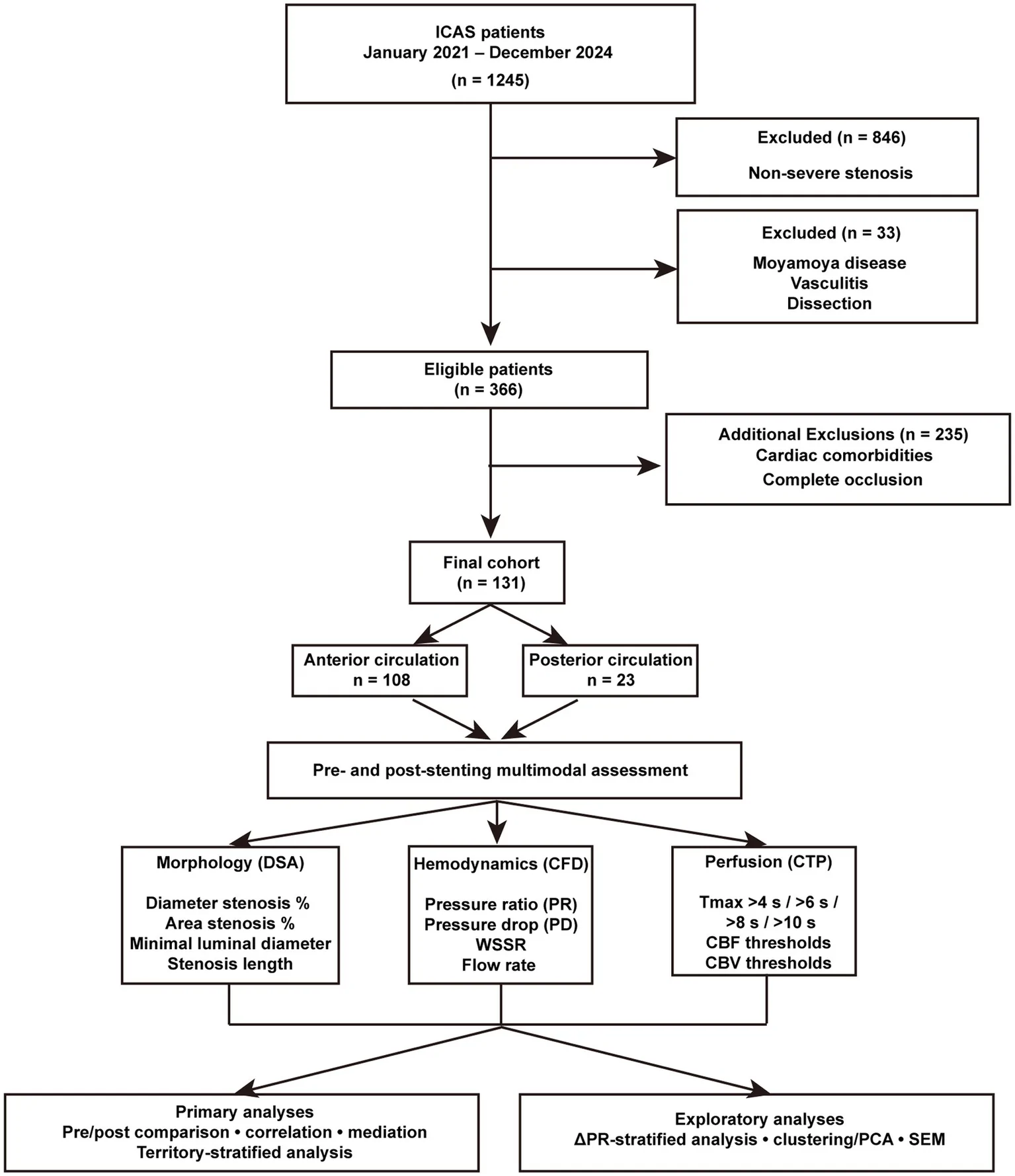
Study flowchart and multimodal analysis framework. This flowchart illustrates the patient selection process and the overall study design. A total of 1245 patients were assessed for eligibility in this retrospective study of intracranial artery stenting conducted at the First Affiliated Hospital of Soochow University between January 2021 and December 2024. After exclusion of patients with non-atherosclerotic stenosis, complete occlusion, cardiac comorbidities, or inadequate imaging, 131 patients with severe intracranial atherosclerotic stenosis (70–99%) were included. All included patients underwent intracranial stenting and multimodal evaluation. Vascular morphology was assessed using digital subtraction angiography, hemodynamics was quantified using computational fluid dynamics, and CT perfusion metrics were used to evaluate perfusion imaging changes. Primary and exploratory analyses were subsequently performed to characterize morphology–hemodynamics–perfusion relationships after stenting.

A total of 131 patients were included in the final analysis (Figure 1), with 108 lesions in the anterior circulation and 23 lesions in the posterior circulation. Baseline demographic, clinical, laboratory, and neurological characteristics are summarized in Table 1. No significant differences were observed between the two groups in age, blood pressure, metabolic indices (fasting glucose, lipid profiles, and HbA1c), or neurological severity at admission assessed by mRS and NIHSS scores (all *p* > 0.05). The prevalence of major vascular risk factors, including sex distribution, smoking, dyslipidemia, hypertension, diabetes mellitus, ischemic heart disease, and prior stroke or transient ischemic attack, was also comparable between groups (all *p* > 0.05). Overall, baseline characteristics were broadly comparable between patients with anterior and posterior circulation stenosis.

**Table 1:** Baseline characteristics of the study population.

Variable	Anterior circulation (*n* = 108)	Posterior circulation (*n* = 23)	*p*-value
Age (years)	65.50 [57.00, 69.00]	67.00 [63.50, 70.00]	0.053
Sex (male)	77 (71.3%)	20 (87.0%)	0.196
Systolic blood pressure (mmHg)	138.72 ± 18.89	139.65 ± 18.19	0.826
Diastolic blood pressure (mmHg)	79.00 ± 10.71	81.48 ± 9.56	0.277
Fasting glucose (mmol/L)	5.22 [4.69, 5.96]	5.40 [4.87, 6.65]	0.302
Triglycerides (mmol/L)	1.31 [0.91, 1.75]	1.25 [1.04, 1.60]	0.973
HbA1c (%)	5.80 [5.40, 6.30]	6.10 [5.60, 7.35]	0.132
HDL (mmol/L)	0.94 [0.79, 1.11]	0.85 [0.74, 0.99]	0.118
LDL-C (mmol/L)	1.79 [1.33, 2.40]	1.73 [1.23, 2.16]	0.535
Pre-admission mRS	2.00 [0.00, 3.00]	1.00 [0.00, 3.00]	0.477
Admission NIHSS	1.00 [0.00, 3.00]	1.00 [0.00, 4.00]	0.752
Smoking history	24 (22.4%)	6 (26.1%)	0.916
Dyslipidemia history	57 (52.8%)	14 (60.9%)	0.634
Hypertension history	81 (75.0%)	19 (82.6%)	0.610
Diabetes history	33 (30.6%)	11 (47.8%)	0.177
Stroke/TIA history	64 (59.3%)	15 (65.2%)	0.768

### 3.2 Multimodal morphological, hemodynamic and perfusion imaging changes after intracranial stenting

**Figure 2:**
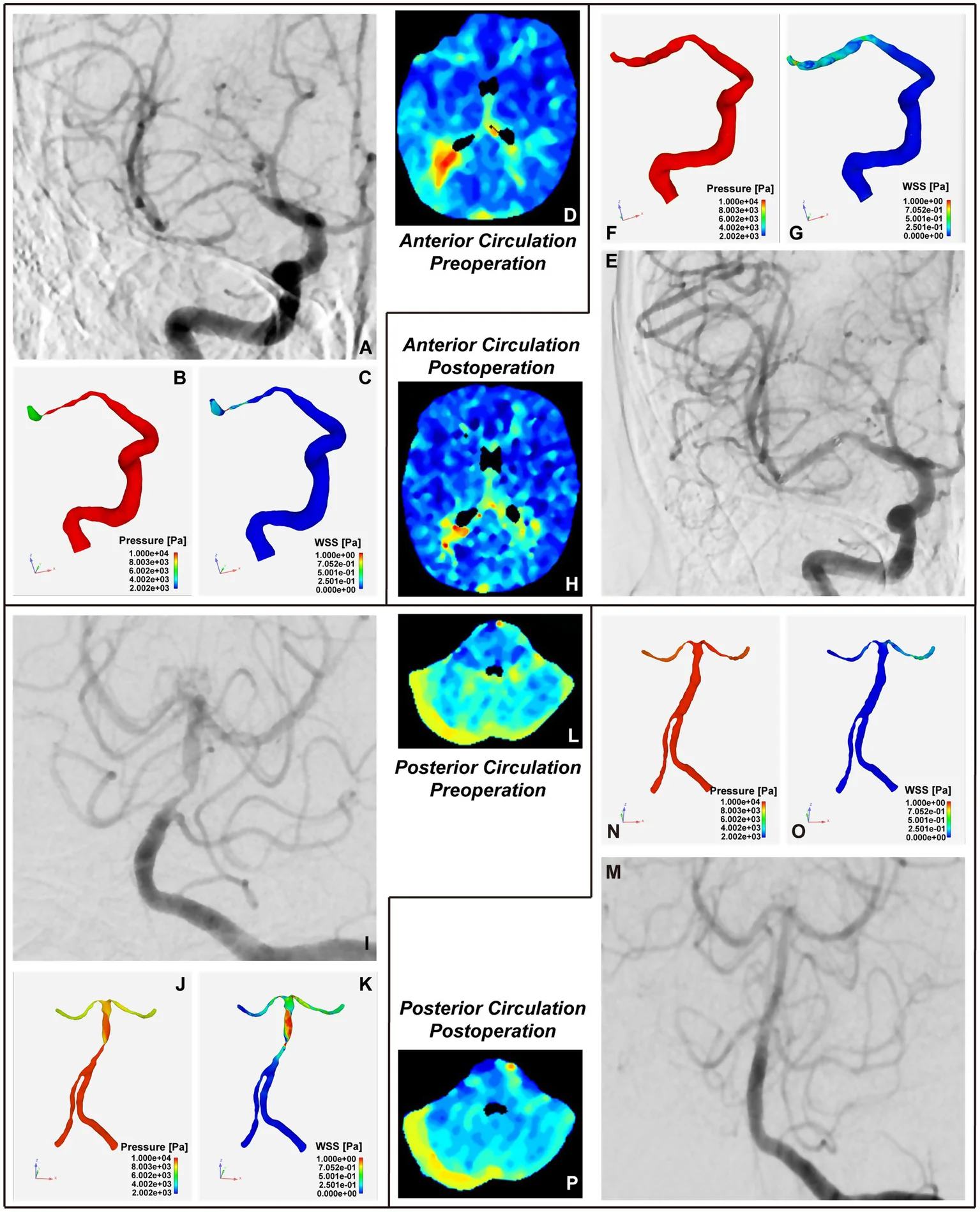
Representative multimodal images before and after intracranial artery stenting in anterior and posterior circulation lesions. Anterior circulation case: before stenting, **(A)** DSA image showing severe right middle cerebral artery stenosis; **(B)** CFD-derived pressure ratio map; **(C)** CFD-derived wall shear stress ratio map; and **(D)** CT perfusion map. After stenting, **(E)** DSA image showing improvement of right middle cerebral artery stenosis; **(F)** increased CFD-derived pressure ratio; **(G)** reduced wall shear stress ratio; and **(H)** CT perfusion map showing improvement in perfusion-delay abnormalities. Posterior circulation case: before stenting, **(I)** DSA image showing severe basilar artery stenosis; **(J)** CFD-derived pressure ratio map; **(K)** CFD-derived wall shear stress ratio map; and **(L)** CT perfusion map. After stenting, **(M)** DSA image showing improvement of basilar artery stenosis; **(N)** increased CFD-derived pressure ratio; **(O)** reduced wall shear stress ratio; and **(P)** CT perfusion map showing improvement in perfusion-delay abnormalities.

**Figure 3:**
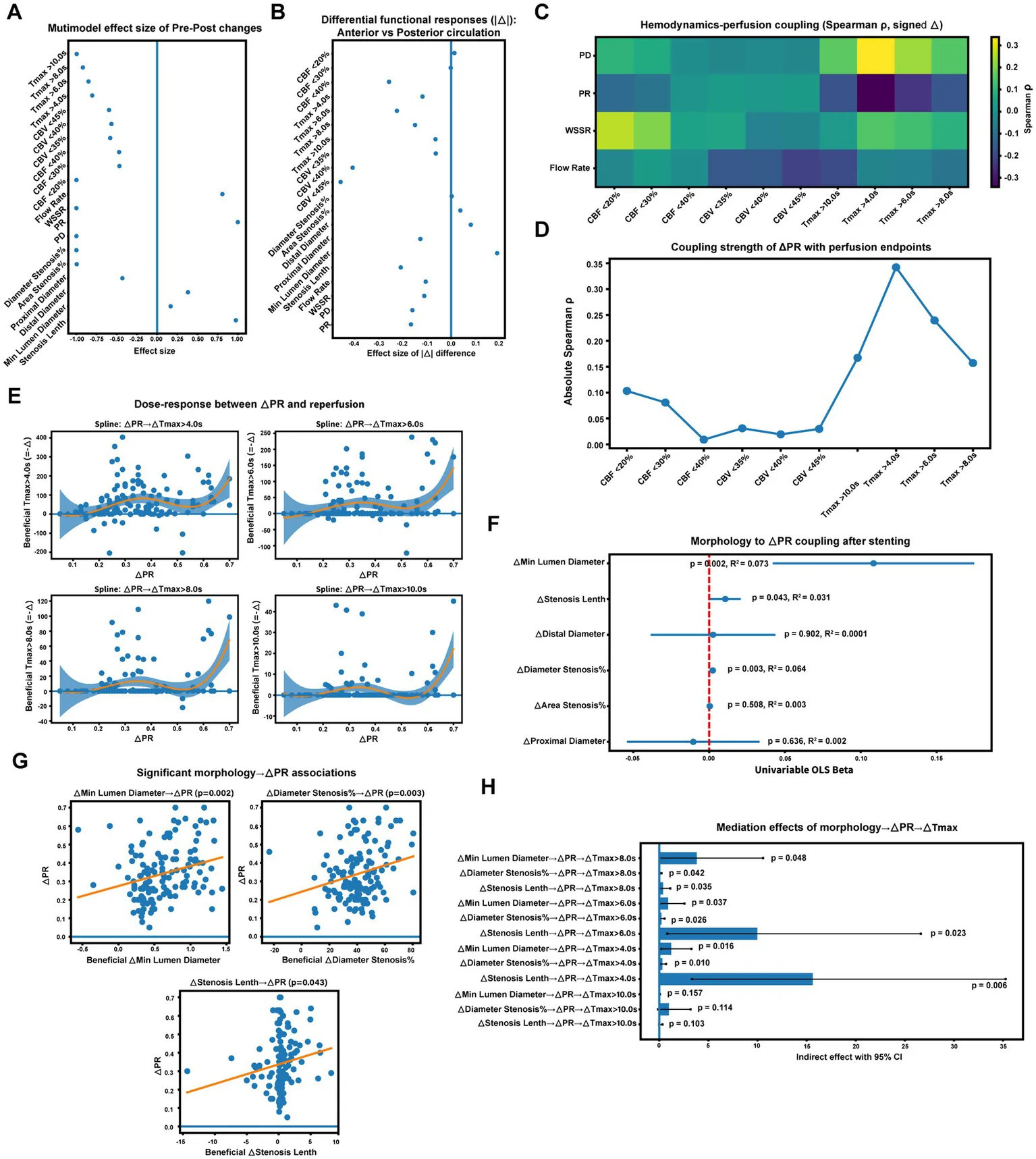
Multimodal characterization of morphology–hemodynamics–perfusion relationships after intracranial stenting. **(A)** Standardized effect sizes of morphological, hemodynamic, and perfusion imaging changes after intracranial stenting. **(B)** Comparison of absolute change magnitudes (|Δ|) between anterior and posterior circulation after stenting. **(C)** Correlation heatmap of hemodynamic and CT perfusion changes. **(D)** Associations between ΔPR and perfusion-delay improvement. **(E)** Nonlinear association between ΔPR and ΔTmax. **(F)** Associations between morphological changes and ΔPR after stenting. **(G)** Scatter plots of significant morphology–hemodynamic associations. **(H)** Mediation effects of hemodynamic changes on the morphology–perfusion association after intracranial stenting.

At the approximately 6-month follow-up, intracranial stenting was associated with significant multimodal alterations in vascular morphology, hemodynamic parameters, and CT perfusion metrics, with overall treatment effects summarized by standardized effect sizes (Figure 2; Table 2). Stenting markedly reconstructed the stenotic segment, as evidenced by reduced diameter and area stenosis percentages, increased minimal luminal diameters, and shortened stenosis length, indicating effective restoration of vessel patency. Concurrently, hemodynamic changes were characterized by increased flow rate in the stenosis-bearing artery, reduced pressure drop and wall shear stress ratio, and an elevated pressure ratio, consistent with diminished translesional pressure loss. Correspondingly, Tmax-based perfusion-delay volumes (>4.0–10.0 s) were significantly reduced following stenting. Changes were also observed in threshold-based cerebral blood flow (CBF) and cerebral blood volume (CBV) abnormality volumes; however, these metrics exhibited markedly zero-inflated distributions and were therefore interpreted with caution. Effect size analysis revealed a coherent multimodal response, with the largest effects observed for stenosis relief and hemodynamic improvements, followed by changes in perfusion imaging metrics (Figure 3A). Stratified effect size analyses identified distinct territory-related patterns (Figure 3B; Table 3): morphological changes were broadly comparable between the anterior and posterior circulations; in contrast, hemodynamic and perfusion imaging changes demonstrated greater territorial heterogeneity. The most pronounced anterior-favoring differences were observed for Tmax-based perfusion-delay metrics at intermediate thresholds (>4–8 s).

**Table 2:** Morphological, hemodynamic, and perfusion changes before and after intracranial stent implantation.

Variable	All (anterior circulation +posterior circulation)			Anterior circulation			Posterior circulation		
	Pre-stenting	Post-stenting	*p*-value	Pre-stenting	Post-stenting	*p*-value	Pre-stenting	Post-stenting	*p*-value
Minimal luminal diameters (mm)	0.81 (0.57–1.04)	1.44 (1.22–1.69)	<0.001	0.78 (0.56–0.98)	1.35 (1.21–1.68)	<0.001	0.96 ± 0.37	1.62 ± 0.33	<0.001
Distal diameters (mm)	2.39 (2.01–2.89)	2.45 (2.02–2.80)	0.096	2.33 (1.95–2.87)	2.37 (1.92–2.77)	0.119	2.75 ± 0.66	2.77 ± 0.57	0.840
Proximal diameters (mm)	2.00 (1.75–2.39)	2.17 (1.78–2.51)	<0.001	1.96 (1.68–2.39)	2.05 (1.73–2.47)	0.001	2.27 ± 0.66	2.42 ± 0.53	0.100
Stenosis length (mm)	5.13 (3.61–7.47)	4.30 (2.96–7.86)	<0.001	5.19 (3.68–7.57)	4.28 (2.92–7.94)	<0.001	4.58 (3.04–6.87)	4.70 (3.86–7.45)	0.345
Diameter stenosis (%)	65.28 (59.47–74.47)	23.45 (13.50–35.73)	<0.001	66.70 (59.22–75.77)	24.13 (13.50–35.73)	<0.001	63.54 ± 9.86	23.81 ± 13.19	<0.001
Area stenosis (%)	86.56 (81.75–92.22)	40.94 (22.50–58.64)	<0.001	86.67 (81.75–92.30)	42.10 (22.48–58.64)	<0.001	86.00 (81.25–89.45)	35.70 (22.83–52.40)	<0.001
PD	37.35 (29.70–47.25)	7.20 (5.40–10.80)	<0.001	37.80 (30.60–48.83)	7.65 (5.40–10.80)	<0.001	32.40 (29.25–39.15)	7.20 (5.85–9.45)	<0.001
PR	0.58 (0.47–0.67)	0.92 (0.88–0.94)	<0.001	0.58 (0.46–0.66)	0.92 (0.88–0.94)	<0.001	0.64 (0.56–0.68)	0.92 (0.90–0.94)	<0.001
WSSR	29.54 (17.94–54.86)	5.94 (3.30–9.88)	<0.001	29.81 (16.99–56.95)	5.84 (3.15–9.69)	<0.001	27.46 (19.25–40.65)	6.75 (3.32–12.81)	<0.001
Flow rate (ml/s)	1.20 (0.67–1.94)	2.27 (1.70–3.06)	<0.001	1.10 (0.66–1.84)	2.20 (1.70–3.06)	<0.001	1.70 (0.79–2.44)	2.51 (1.97–3.21)	<0.001
CBF<20%	0.00 (0.00–0.00)	0.00 (0.00–0.00)	0.005	0.00 (0.00–0.00)	0.00 (0.00–0.00)	0.012	0.00 (0.00–0.00)	0.00 (0.00–0.00)	0.180
CBF<30%	0.00 (0.00–0.00)	0.00 (0.00–0.00)	0.019	0.00 (0.00–0.00)	0.00 (0.00–0.00)	0.024	0.00 (0.00–0.00)	0.00 (0.00–0.00)	0.345
CBF<40%	0.00 (0.00–15.97)	0.00 (0.00–15.45)	0.001	0.00 (0.00–20.43)	0.00 (0.00–17.05)	0.004	0.00 (0.00–0.19)	0.00 (0.00–0.00)	0.128
CBV<35%	0.00 (0.00–0.00)	0.00 (0.00–0.00)	0.007	0.00 (0.00–0.00)	0.00 (0.00–0.00)	0.008	0.00 (0.00–0.00)	0.00 (0.00–0.00)	0.465
CBV<40%	0.00 (0.00–7.20)	0.00 (0.00–2.95)	<0.001	0.28 (0.00–7.67)	0.00 (0.00–4.10)	<0.001	0.00 (0.00–0.02)	0.00 (0.00–0.00)	0.249
CBV<45%	3.65 (0.00–35.75)	2.95 (0.00–14.70)	<0.001	8.50 (0.00–40.55)	8.15 (0.00–20.43)	<0.001	0.00 (0.00–4.35)	0.00 (0.00–1.95)	0.314
Tmax>4.0 s	75.55 (15.85–199.57)	23.95 (4.20–93.88)	<0.001	88.70 (12.75–209.20)	23.30 (4.00–92.20)	<0.001	50.00 (19.55–165.95)	45.70 (9.15–108.05)	0.008
Tmax>6.0 s	0.00 (0.00–79.48)	0.00 (0.00–8.12)	<0.001	0.00 (0.00–95.33)	0.00 (0.00–11.20)	<0.001	0.00 (0.00–0.00)	0.00 (0.00–2.00)	0.046
Tmax>8.0 s	0.00 (0.00–9.02)	0.00 (0.00–0.00)	<0.001	0.00 (0.00–10.12)	0.00 (0.00–0.00)	<0.001	0.00 (0.00–0.00)	0.00 (0.00–0.00)	0.068
Tmax>10.0 s	0.00 (0.00–0.00)	0.00 (0.00–0.00)	<0.001	0.00 (0.00–0.00)	0.00 (0.00–0.00)	<0.001	0.00 (0.00–0.00)	0.00 (0.00–0.00)	0.109

**Table 3:** Comparison of the degree of functional remodeling between anterior and posterior circulations after stenting.

Variable	Anterior circulation	Posterior circulation	*p*-value	q-FDR
ΔMinimal luminal diameters (mm)	0.495 (0.330–0.848)	0.670 (0.455–0.850)	0.153	0.489
ΔDistal diameters (mm)	0.200 (0.068–0.440)	0.080 (0.030–0.450)	0.338	0.660
ΔProximal diameters (mm)	0.185 (0.080–0.495)	0.190 (0.065–0.480)	0.990	0.990
ΔStenosis length (mm)	0.580 (0.200–1.920)	0.300 (0.150–0.990)	0.116	0.466
ΔDiameter stenosis (%)	40.875 ± 13.669	39.730 ± 15.590	0.747	0.916
ΔArea stenosis (%)	46.150 (27.508–61.903)	47.900 (29.805–62.120)	0.778	0.916
ΔPD	29.700 (21.600–38.700)	25.200 (20.250–30.600)	0.227	0.505
ΔPR	0.330 (0.240–0.430)	0.280 (0.225–0.340)	0.210	0.505
ΔWSSR	21.155 (11.630–44.691)	17.000 (13.723–34.377)	0.402	0.660
ΔFlow rate (ml/s)	1.265 (0.602–1.705)	1.000 (0.645–1.455)	0.429	0.660
ΔCBF<20%	0.000 (0.000–0.000)	0.000 (0.000–0.000)	0.833	0.926
ΔCBF<30%	0.000 (0.000–0.002)	0.000 (0.000–0.200)	0.987	0.990
ΔCBF<40%	0.383 (0.000–17.822)	0.000 (0.000–4.688)	0.038	0.255
ΔCBV<35%	0.000 (0.000–0.000)	0.000 (0.000–0.000)	0.505	0.673
ΔCBV<40%	1.300 (0.000–4.725)	0.000 (0.000–0.024)	0.001	0.012
ΔCBV<45%	7.400 (0.000–26.414)	0.000 (0.000–2.700)	<0.001	0.008
ΔTmax>4.0 s	49.800 (5.400–125.650)	25.000 (7.650–60.000)	0.372	0.660
ΔTmax>6.0 s	0.000 (0.000–47.875)	0.000 (0.000–2.000)	0.062	0.309
ΔTmax>8.0 s	0.000 (0.000–8.000)	0.000 (0.000–0.000)	0.171	0.489
ΔTmax>10.0 s	0.000 (0.000–0.000)	0.000 (0.000–0.000)	0.478	0.673

### 3.3 Relationship between morphology, hemodynamics and CT perfusion after stenting

#### 3.3.1 Associations among morphological, hemodynamic and perfusion imaging changes

Correlation analyses revealed structured associations between hemodynamic changes and perfusion imaging changes following intracranial stenting, with ΔPR and ΔPD consistently correlated with Tmax-based perfusion-delay volumes, whereas shear-related indices exhibited weaker and more heterogeneous relationships (Figures 3C,D). Among hemodynamic metrics, ΔPR demonstrated the strongest associations with reductions in Tmax-based perfusion-delay volume at thresholds exceeding 4–8 s (all *p* < 0.05) (Table 4). Spline/GAM analyses further identified a nonlinear but monotonic association pattern, with the most pronounced reductions in Tmax-based perfusion-delay volume observed at lower to intermediate delay thresholds; however, these associations appeared attenuated at the Tmax >10.0 s threshold (Figure 3E). Morphological changes were significantly associated with hemodynamic alterations: improvements in minimum luminal diameter, diameter stenosis percentage, and stenosis length were positively correlated with ΔPR, whereas other geometric parameters showed no significant association (Figures 3F,G). In contrast, morphological parameters alone were not independently associated with changes in Tmax-based perfusion-delay metrics across thresholds (all *p* > 0.05; Table 4). In combined models, the associations of morphological variables with Tmax-based perfusion-delay metrics lost significance after adjustment for ΔPR; meanwhile, ΔPR remained a consistent predictor at Tmax thresholds of >4–8 s. Neither morphological nor hemodynamic variables demonstrated robust associations at the Tmax >10.0 s threshold (Table 4). Collectively, these findings suggested a sequential statistical pattern in which morphological changes were associated with hemodynamic alterations, particularly ΔPR, which in turn were linked to changes in Tmax-based perfusion-delay metrics. These associations were most evident at lower to intermediate delay thresholds and less pronounced at the Tmax >10.0 s threshold.

**Table 4:** Comparative analysis of differences under varying morphological, hemodynamic, and perfusion pathways.

Model path	ΔTmax>4.0 s				ΔTmax>6.0 s				ΔTmax>8.0 s				ΔTmax>10.0 s			
	β	CI-low	CI-high	*p-*value	β	CI-low	CI-high	*p*-value	β	CI-low	CI-high	*p*-value	β	CI-low	CI-high	*p*-value
A: ΔMinimal luminal diameters (mm) → ΔPR	0.109	0.043	0.175	0.001	0.109	0.043	0.175	0.001	0.109	0.043	0.175	0.001	0.109	0.043	0.175	0.001
A: ΔDiameter stenosis (%) → ΔPR	0.002	0.001	0.004	0.003	0.002	0.001	0.004	0.003	0.002	0.001	0.004	0.003	0.002	0.001	0.004	0.003
A: ΔStenosis length (mm) → ΔPR	0.011	0.000	0.021	0.046	0.011	0.000	0.021	0.046	0.011	0.000	0.021	0.046	0.011	0.000	0.021	0.046
B: ΔPR → Perfusion	126.084	23.430	228.737	0.016	92.330	25.867	158.794	0.007	38.199	10.400	65.998	0.007	8.995	−0.091	18.081	0.052
C: ΔMinimal luminal diameters (mm) → Perfusion	−10.010	−52.245	32.225	0.640	11.133	−16.328	38.594	0.424	8.606	−2.807	20.020	0.138	1.130	−2.579	4.839	0.548
C: ΔDiameter stenosis (%) → Perfusion	0.086	−0.874	1.046	0.860	0.090	−0.535	0.715	0.776	0.096	−0.165	0.357	0.467	0.020	−0.064	0.105	0.634
C: ΔStenosis length (mm) → Perfusion	5.376	−1.038	11.790	0.100	3.558	−0.618	7.734	0.094	0.975	−0.780	2.731	0.274	0.084	−0.485	0.653	0.771
D: ΔMinimal luminal diameters (mm) → ΔPR (adjusted) → Perfusion	−25.633	−68.557	17.291	0.240	1.150	−26.784	29.084	0.935	4.792	−6.863	16.447	0.418	0.161	−3.658	3.980	0.934
D: ΔMinimal luminal diameters (mm) → ΔPR (unadjusted) → Perfusion	143.283	36.802	249.763	0.009	91.559	22.263	160.854	0.010	34.983	6.070	63.897	0.018	8.887	−0.586	18.360	0.066
D: ΔDiameter stenosis (%) → ΔPR (adjusted) → Perfusion	−0.221	−1.196	0.754	0.654	−0.133	−0.764	0.498	0.678	0.008	−0.256	0.272	0.951	−0.001	−0.087	0.086	0.989
D: ΔDiameter stenosis (%) → ΔPR (unadjusted) → Perfusion	132.206	25.764	238.647	0.015	96.006	27.082	164.930	0.007	37.973	9.127	66.819	0.010	9.011	−0.417	18.440	0.061
D: ΔStenosis length (mm) → ΔPR (adjusted) → Perfusion	4.171	−2.251	10.592	0.201	2.664	−1.495	6.822	0.207	0.591	−1.156	2.337	0.505	−0.011	−0.583	0.561	0.970
D: ΔStenosis length (mm) → ΔPR (unadjusted) → Perfusion	114.593	10.661	218.526	0.031	84.991	17.688	152.294	0.014	36.571	8.301	64.841	0.012	9.025	−0.230	18.280	0.056

#### 3.3.2 Mediation effects of hemodynamic changes on the morphology-perfusion association

Bootstrap mediation analyses (5,000 resamples) revealed that hemodynamic changes, particularly ΔPR, exerted a significant mediating effect on the association between morphological remodeling and perfusion imaging changes following stenting (Figure 3H; Table 5). Significant indirect effects were observed for key morphological parameters, specifically increases in minimal luminal diameter and reductions in diameter stenosis percentage, on improvements in Tmax-based perfusion-delay metrics at thresholds exceeding 4–8 s, with bootstrap 95% confidence intervals excluding zero. The mediating effects exhibited a threshold-dependent pattern, demonstrating stronger effects at lower to intermediate delay thresholds and attenuated effects at the Tmax >10 s threshold. Subgroup analyses indicated that these mediating effects were present in the anterior circulation but not evident in the posterior circulation, suggesting territorial heterogeneity in the morphology-hemodynamic-perfusion association.

**Table 5:** Bootstrap mediation analysis of ΔPR in the association between morphological remodeling and Tmax-based perfusion-delay improvement after intracranial stenting.

Model path	All (Anterior Circulation+Posterior Circulation)				Anterior Circulation			
	Indirect	Indirect CI-low	Indirect CI-high	P-indirect	Indirect	Indirect CI-low	Indirect CI-high	P-indirect
ΔMinimal luminal diameters (mm) → ΔPR → ΔTmax>4.0 s	15.623	3.326	35.269	0.006	13.789	0.713	33.892	0.031
ΔDiameter stenosis (%) → ΔPR → ΔTmax>4.0 s	0.307	0.054	0.690	0.010	0.297	0.006	0.749	0.042
ΔStenosis length (mm) → ΔPR → ΔTmax>4.0 s	1.205	0.180	3.276	0.016	1.399	−0.050	3.856	0.056
ΔMinimal luminal diameters (mm) → ΔPR → Tmax>6.0 s	9.983	0.801	26.630	0.023	8.737	−0.327	26.167	0.066
ΔDiameter stenosis (%) → ΔPR → Tmax>6.0 s	0.223	0.021	0.543	0.026	0.218	−0.011	0.597	0.064
ΔStenosis length (mm) → ΔPR → Tmax>6.0 s	0.894	0.041	2.557	0.037	1.046	−0.128	3.151	0.086
ΔMinimal luminal diameters (mm) → ΔPR → Tmax>8.0 s	3.814	0.009	10.567	0.048	3.057	−0.439	9.622	0.110
ΔDiameter stenosis (%) → ΔPR → Tmax>8.0 s	0.088	0.002	0.227	0.042	0.076	−0.012	0.222	0.106
ΔStenosis length (mm) → ΔPR → Tmax>8.0 s	0.385	0.017	1.122	0.035	0.419	−0.037	1.315	0.085
ΔMinimal luminal diameters (mm) → ΔPR → Tmax>10.0 s	0.969	−0.131	3.182	0.114	0.422	−0.358	1.876	0.348
ΔDiameter stenosis (%) → ΔPR → Tmax>10.0 s	0.021	−0.006	0.064	0.157	0.009	−0.017	0.040	0.470
ΔStenosis length (mm) → ΔPR → Tmax>10.0 s	0.095	−0.011	0.323	0.103	0.059	−0.048	0.239	0.302

### 3.4 Exploratory multivariable patterns across morphology, hemodynamics and perfusion imaging

**Figure 4:**
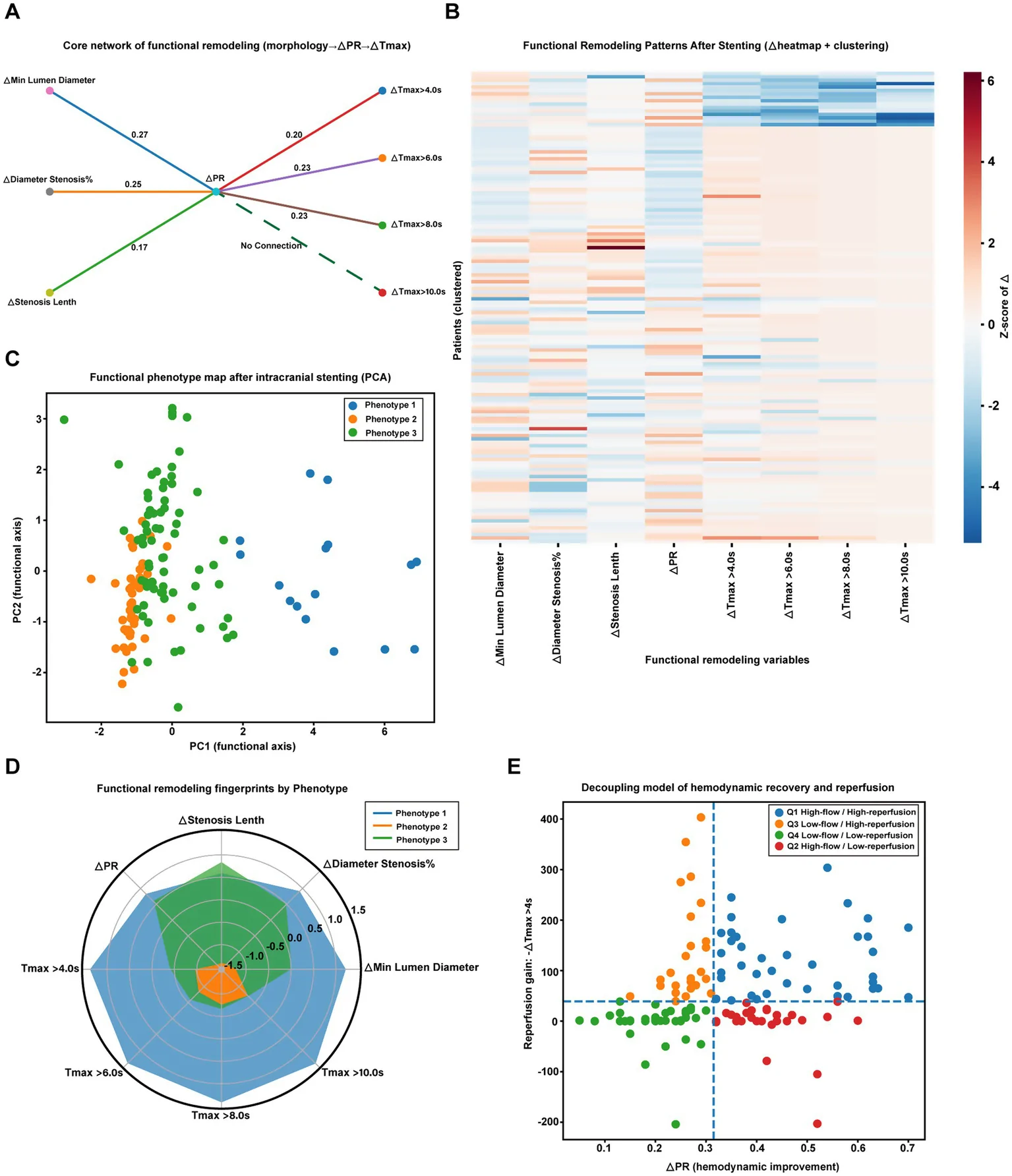
Exploratory multivariable patterns of morphology–hemodynamics–perfusion changes after intracranial stenting. **(A)** Network diagram of morphology–hemodynamics–perfusion associations centered on ΔPR. **(B)** Unsupervised clustering of multimodal post-stenting change patterns. **(C)** Principal component analysis (PCA) of integrated morphology–hemodynamics–perfusion variation after stenting. **(D)** Radar plots illustrating the multidimensional profiles of the three exploratory response patterns. **(E)** Exploratory stratification of hemodynamic and perfusion imaging changes after intracranial stenting.

Exploratory integration of morphological remodeling, hemodynamic changes (particularly ΔPR), and Tmax-based perfusion-delay changes suggested a hemodynamics-centered multivariable association pattern following intracranial stenting. Network analysis revealed that ΔPR occupied a central position within the observed morphology-hemodynamic-perfusion association network and exhibited stronger connectivity with both morphological variables and Tmax-based perfusion-delay changes than other hemodynamic indices (Figure 4A). Among morphological parameters, improvement in minimal luminal diameter showed the strongest association with ΔPR, followed by reductions in diameter stenosis percentage and stenosis length; meanwhile, ΔPR was significantly associated with reductions in Tmax-based perfusion-delay volumes at thresholds exceeding 4–8 s, consistent with its relevance as an informative hemodynamic marker of perfusion-delay change. Unsupervised clustering of standardized multimodal Δ-features identified distinct multivariable change patterns (Figure 4B), while principal component analysis summarized the major dimensions of integrated morphology–hemodynamic–perfusion variation (Figure 4C), enabling stratification into three exploratory response patterns. Pattern 1 demonstrated coordinated morphological remodeling, marked ΔPR changes, and consistent reductions in Tmax-based perfusion-delay volume across thresholds; Pattern 2 exhibited limited changes across all domains; and Pattern 3 displayed intermediate morphological and hemodynamic changes alongside attenuated perfusion-delay improvement. Radar plots further illustrated these multidimensional change profiles (Figure 4D). Collectively, these exploratory analyses suggest heterogeneous multivariable change patterns following stenting, with ΔPR remaining closely aligned with both morphological variables and Tmax-based perfusion-delay changes.

### 3.5 Territory-specific differences in morphology–hemodynamic–perfusion change patterns

To further characterize post-stenting morphology–hemodynamic–perfusion change patterns, exploratory indices describing the relationships from morphological remodeling to hemodynamic change and from hemodynamic change to perfusion imaging change were evaluated (Table 6). The exploratory morphology-to-hemodynamics efficiency index (MHE) exhibited circulation-specific variation, with higher diameter-based values in the anterior circulation and greater sensitivity to stenosis length reduction in the posterior circulation. The exploratory hemodynamics-to-perfusion efficiency index (HPE) indicated weaker hemodynamic–perfusion correspondence in the posterior circulation, showing less pronounced reductions in Tmax-based perfusion-delay volume at higher delay thresholds (Tmax >6–10 s), whereas the exploratory morphology-to-perfusion index (MPE) remained relatively higher in the anterior circulation. Pattern-based analyses further identified three exploratory multimodal response patterns (Table 6): one characterized by coordinated morphological remodeling, marked ΔPR change, and consistent reductions in Tmax-based perfusion-delay metrics; one with limited changes across all domains; and one featuring intermediate morphological and hemodynamic changes but attenuated perfusion-delay improvement. An exploratory stratification based on ΔPR and changes in Tmax >4.0 s volume (Figure 4E) further revealed heterogeneous response groups, including concordant high-response, hemodynamic-dominant, perfusion-dominant, and globally low-response patterns. These findings further suggest heterogeneity in the relationship between hemodynamic change and perfusion imaging change after stenting, particularly across vascular territories.

**Table 6:** Efficiency metrics of morpho-hemodynamic-perfusion coupling at different positions and functional phenotypes after stenting.

Coupling type	Model path	Anterior + Posterior Circulation	Anterior circulation	Posterior circulation	Phenotype 1	Phenotype 2	Phenotype 3
Morphology-to-hemodynamics (MHE)	ΔMinimal luminal diameters (mm) → ΔPR	0.529 [0.365, 0.765]	0.564 [0.388, 0.787]	0.443 [0.323, 0.58]	0.63 [0.382, 0.777]	0.495 [0.301, 0.682]	0.574 [0.385, 0.824]
	ΔDiameter stenosis (%) → ΔPR	0.00789 [0.00563, 0.0112]	0.00798 [0.0056, 0.0112]	0.00751 [0.00618, 0.0103]	0.00841 [0.00666, 0.0124]	0.00589 [0.00445, 0.00798]	0.00944 [0.00696, 0.0117]
	ΔStenosis length (mm) → ΔPR	0.42 [0.0617, 1.37]	0.369 [0.0635, 1.34]	0.867 [0.0301, 2.14]	1.57 [0.343, 4.08]	0.278 [−0.0924, 1.19]	0.379 [0.124, 1.34]
Hemodynamics-to-perfusion (HPE)	ΔPR → ΔTmax>4.0 s	100 [8.67, 289]	105 [8.75, 293]	57.1 [13.3, 259]	482 [316, 777]	23.9 [0, 86.3]	126 [26.5, 290]
	ΔPR → ΔTmax>6.0 s	0 [0, 111]	0 [0, 125]	0 [0, 0]	406 [296, 493]	0 [0, 0]	0 [0, 94.3]
	ΔPR → ΔTmax>8.0 s	0 [0, 12.7]	0 [0, 13.8]	0 [0, 0]	144 [120, 223]	0 [0, 0]	0 [0, 9.11]
	ΔPR → ΔTmax>10.0 s	0 [0, 0]	0 [0, 0]	0 [0, 0]	31.1 [19.3, 66.7]	0 [0, 0]	0 [0, 0]
Morphology-to-perfusion (MPE)	ΔMinimal luminal diameters (mm) → ΔTmax>4.0 s	48.7 [2.77, 155]	52 [3.05, 180]	27.7 [0.941, 83.4]	261 [182, 431]	11 [0, 48.4]	57.9 [12.2, 155]
	ΔDiameter stenosis (%) → ΔTmax>4.0 s	0.779 [0.047, 2.37]	0.821 [0.044, 2.74]	0.409 [0.13, 1.27]	4.98 [3.33, 6.01]	0.117 [0, 0.455]	0.995 [0.206, 2.32]
	ΔStenosis length (mm) → ΔTmax>4.0 s	23.3 [0, 228]	22.1 [0, 184]	25.5 [0.111, 308]	581 [185, 2,820]	0 [−2.53, 29.9]	36 [−0, 208]

### 3.6 ΔPR-stratified patterns of Tmax-based perfusion-delay change

**Figure 5:**
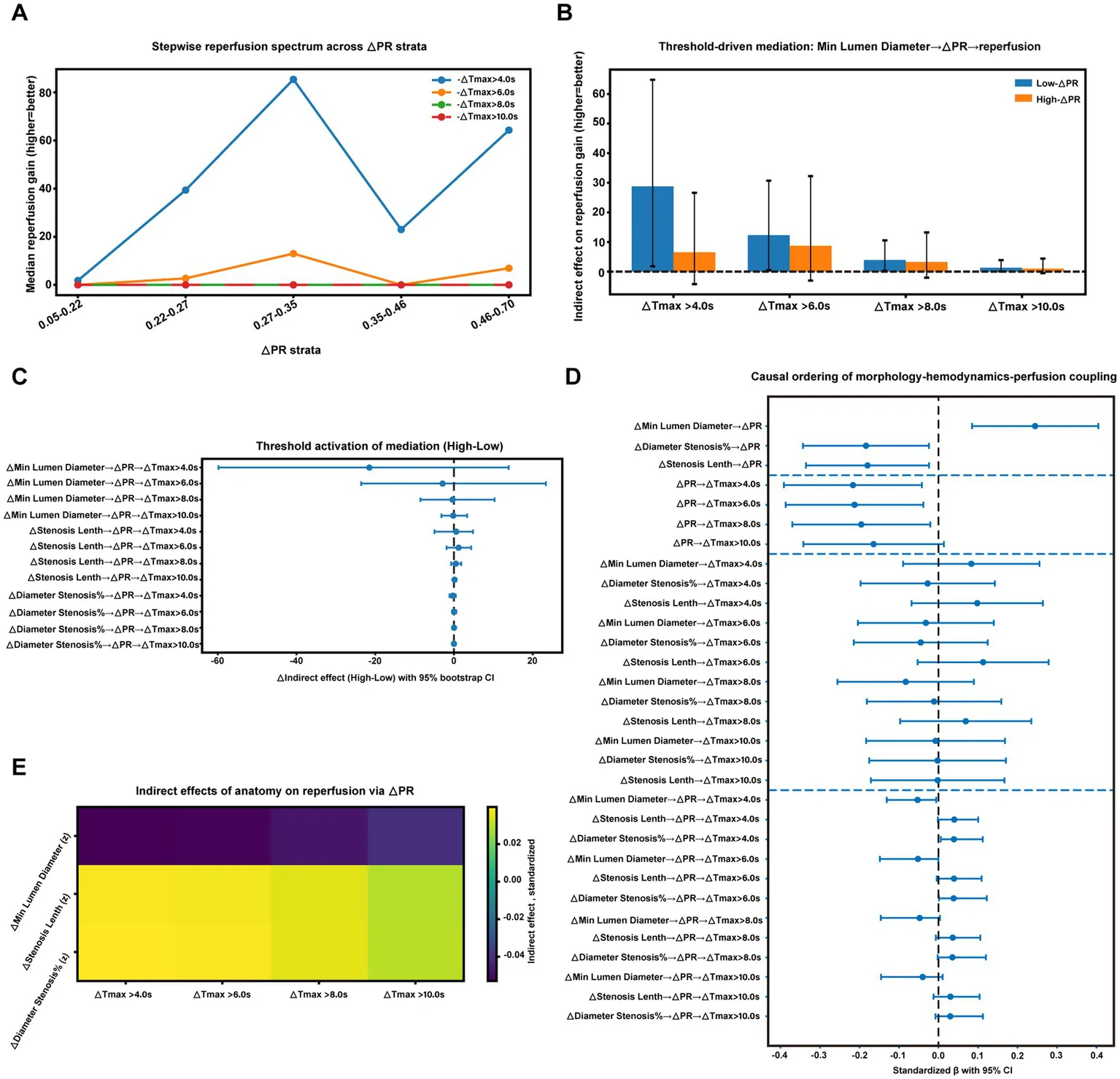
ΔPR-stratified patterns of perfusion-delay change and exploratory mediation/SEM analyses after intracranial stenting. **(A)** Stepwise pattern of Tmax-based perfusion-delay change across ΔPR strata. **(B)** Threshold-stratified serial mediation analysis of morphological remodeling, ΔPR, and perfusion-delay change. **(C)** Comparison of indirect effects between low- and high-ΔPR strata. **(D)** Exploratory serial pathway and structural equation modeling of morphology–hemodynamics–perfusion associations after intracranial stenting. **(E)** Heatmap of indirect effects (a × b) for anatomical variables associated with ΔTmax change.

To facilitate interpretation of the continuous association pattern, a ΔPR-stratified profile of Tmax-based perfusion-delay change was constructed (Figure 5A). Across five ΔPR quantiles, median reductions in Tmax-based perfusion-delay volume (−ΔTmax) exhibited a stepwise pattern, with more evident changes observed for Tmax >4.0 s and >6.0 s as ΔPR increased; in contrast, changes at Tmax >8.0 s and >10.0 s remained limited, suggesting weaker associations at higher delay thresholds. A visible inflection range was observed at ΔPR = 0.27–0.35, suggesting a possible nonlinear transition zone in the association between ΔPR and Tmax-based perfusion-delay change. Stratified bootstrap serial mediation analyses further suggested that the indirect association between morphological remodeling and Tmax-based perfusion-delay change varied across ΔPR strata (Figure 5B). In the low ΔPR stratum (ΔPR < 0.31), improvement in minimal luminal diameter showed significant indirect effects on Tmax-based perfusion-delay metrics through ΔPR at thresholds of >4–8 s, indicating a significant serial mediation pattern involving Δminimum luminal diameter, ΔPR, and ΔTmax. These indirect effects were no longer significant in the high-ΔPR stratum, suggesting attenuation of the association at higher ΔPR levels. Contrasts of indirect effects are presented in Figure 5C. Point estimates for minimal luminal diameter tended to favor the low-ΔPR stratum at Tmax >4–8 s, although bootstrap confidence intervals overlapped, suggesting possible heterogeneity. In contrast, indirect effects related to stenosis length and diameter stenosis percentage remained limited across thresholds.

### 3.7 Exploratory structural equation modeling of morphology–hemodynamic–perfusion associations

Structural equation modeling indicated that the association between morphological remodeling and perfusion imaging changes following stenting could be described by a limited number of statistically coherent pathways. Increases in minimal luminal diameter, along with reductions in stenosis length and diameter stenosis percentage, were consistently associated with hemodynamic changes, particularly ΔPR (Figure 5D). In turn, ΔPR demonstrated graded associations with changes in Tmax-based perfusion-delay metrics across different thresholds, particularly at the Tmax >6 s and >8 s thresholds. Direct associations between morphological parameters and Tmax-based perfusion-delay changes were weak and largely non-significant after adjusting for ΔPR; however, indirect morphology–ΔPR–perfusion-delay associations remained directionally consistent, as illustrated by the mediation-based heatmap (Figure 5E). Global model fit indices supported the internal consistency of the exploratory SEM framework, with the standardized model demonstrating good fit (CFI and NFI > 0.90, RMSEA < 0.01). These findings suggest that the hemodynamics-centered SEM was broadly consistent with the primary morphology-hemodynamic-perfusion association analyses following stenting.

## 4 Discussion

This study presents an exploratory multimodal analysis of post-stenting changes in vascular morphology, hemodynamics derived from computational fluid dynamics (CFD), and CT perfusion metrics in patients with severe intracranial atherosclerotic stenosis. Although intracranial stenting was associated with consistent improvement in luminal geometry and lesion-level hemodynamic parameters, changes in perfusion imaging metrics could not be fully explained by anatomical remodeling alone. Instead, changes in translesional pressure ratio (ΔPR) demonstrated more consistent associations with Tmax-based perfusion-delay metrics than with geometric stenosis measures, suggesting that pressure recovery may provide additional hemodynamic information beyond morphological stenosis relief alone.

Assessment timing is important when interpreting post-stenting hemodynamic changes. Immediate post-deployment DSA- and CFD-based measurements primarily reflect acute changes in luminal geometry, translational flow, and pressure (24, 25), whereas CTP performed within 3 days (26), at 1 week (14), or at a mean of 5.5 days after treatment (15) mainly characterizes early perfusion responses. By contrast, angiographic and perfusion follow-up at 3–12 months and assessment of in-stent restenosis at 6 months may additionally reflect vascular adaptation and stent-patency status (24, 25). Because our post-stenting DSA and CTA/CTP were obtained at approximately 6 months, the present findings represent medium-term rather than immediate or 24−/48-h hemodynamic and perfusion changes.

Our findings are broadly consistent with previous studies indicating that intracranial stenting can improve luminal geometry and lesion-level hemodynamic parameters in ICAS, as well as with computational fluid dynamics (CFD)-based analyses reporting increased flow and reduced pressure loss following intervention (12, 13, 24, 25, 27, 28). By incorporating CT perfusion metrics, the present study further suggests that anatomical improvement alone does not uniformly correspond to downstream perfusion imaging changes after stenting. In particular, associations between hemodynamic and perfusion imaging changes were more evident in the anterior circulation, whereas these relationships were weaker in the posterior circulation, suggesting territorial heterogeneity that may relate to differences in collateral buffering capacity, downstream vascular resistance, or microvascular reserve (26, 29).

Among the hemodynamic variables examined, ΔPR demonstrated the closest associations with Tmax-based perfusion-delay metrics and exhibited a nonlinear association pattern across thresholds. In contrast, morphological variables alone were not independently associated with Tmax-based perfusion-delay changes after adjustment for ΔPR. These findings suggest that the relationship between anatomical remodeling and perfusion imaging change may be more clearly reflected through hemodynamic alterations, particularly ΔPR, than through geometric measures alone. The additional clustering, stratified, and SEM analyses were generally consistent with this interpretation; however, they should be regarded as exploratory characterizations of multivariable association patterns rather than definitive evidence of a direct mechanistic pathway.

Several limitations should be acknowledged. First, this was a single-center retrospective study, and the relatively small sample size in the posterior circulation may have limited the statistical power for subgroup analyses. Second, long-term clinical outcomes were not incorporated, which limited assessment of the prognostic relevance of the observed imaging and hemodynamic associations. Third, Tmax and CFD-derived pressure metrics should be interpreted cautiously in this chronic ICAS setting, as they reflect perfusion delay and model-based hemodynamic characteristics rather than direct measures of tissue viability or purely lesion-specific physiology. Fourth, because imaging was available only before stenting and at approximately 6 months, we could not characterize immediate, 24−/48-h, or early postprocedural trajectories or separate the direct procedural effect from subsequent vascular and microvascular adaptation. In addition, DSA and CTA/CTP were not always acquired on the same calendar day, although they were scheduled within the same assessment window. Despite these limitations, the present study suggests that ΔPR may provide functionally relevant information beyond anatomical stenosis measures in characterizing post-stenting morphology–hemodynamic–perfusion relationships. Future multicenter prospective studies with standardized serial imaging and longitudinal follow-up are needed to validate these findings, characterize time-dependent post-stenting changes, and clarify their clinical relevance.

## 5 Conclusion

In severe ICAS, post-stenting changes in translesional pressure ratio were more consistently associated with improvement in perfusion-delay metrics than morphological stenosis measures alone. Pressure ratio changes may therefore provide a useful hemodynamic link between anatomical remodeling and downstream perfusion imaging changes.

## Data Availability

The raw data supporting the conclusions of this article will be made available by the authors, without undue reservation.
